# Mesenchymal Stem Cell-Derived Exosomes and Other Extracellular Vesicles as New Remedies in the Therapy of Inflammatory Diseases

**DOI:** 10.3390/cells8121605

**Published:** 2019-12-11

**Authors:** Carl Randall Harrell, Nemanja Jovicic, Valentin Djonov, Nebojsa Arsenijevic, Vladislav Volarevic

**Affiliations:** 1 Regenerative Processing Plant, LLC, 34176 US Highway 19 N Palm Harbor, Palm Harbor, FL 34684, USA; dr.harrell@regenerativeplant.org; 2 Department for Microbiology and Immunology, Center for Molecular Medicine and Stem Cell Research, Faculty of Medical Sciences, University of Kragujevac, 69 Svetozar Markovic Street, 34000 Kragujevac, Serbia; nemanjajovicic.kg@gmail.com (N.J.); arne@medf.kg.ac.rs (N.A.); 3 Institute of Anatomy, University of Bern, 2 Baltzerstrasse, 3012 Bern, Switzerland; valentin.djonov@ana.unibe.ch

**Keywords:** mesenchymal stem cells, extracellular vesicles, therapy, immunosuppression, regeneration

## Abstract

There is growing evidence that mesenchymal stem cell (MSC)-based immunosuppression was mainly attributed to the effects of MSC-derived extracellular vesicles (MSC-EVs). MSC-EVs are enriched with MSC-sourced bioactive molecules (messenger RNA (mRNA), microRNAs (miRNAs), cytokines, chemokines, immunomodulatory factors) that regulate phenotype, function and homing of immune cells. In this review article we emphasized current knowledge regarding molecular mechanisms responsible for the therapeutic effects of MSC-EVs in attenuation of autoimmune and inflammatory diseases. We described the disease-specific cellular targets of MSC-EVs and defined MSC-sourced molecules, which were responsible for MSC-EV-based immunosuppression. Results obtained in a large number of experimental studies revealed that both local and systemic administration of MSC-EVs efficiently suppressed detrimental immune response in inflamed tissues and promoted survival and regeneration of injured parenchymal cells. MSC-EVs-based anti-inflammatory effects were relied on the delivery of immunoregulatory miRNAs and immunomodulatory proteins in inflammatory immune cells (M1 macrophages, dendritic cells (DCs), CD4+Th1 and Th17 cells), enabling their phenotypic conversion into immunosuppressive M2 macrophages, tolerogenic DCs and T regulatory cells. Additionally, through the delivery of mRNAs and miRNAs, MSC-EVs activated autophagy and/or inhibited apoptosis, necrosis and oxidative stress in injured hepatocytes, neurons, retinal cells, lung, gut and renal epithelial cells, promoting their survival and regeneration.

## 1. Introduction

Epidemiological studies revealed a significant increase in the incidence of autoimmune and inflammatory diseases during the last two decades [1]. Accordingly, the total number of patients taking immunosuppressive drugs has been continuously increasing [2]. Long-term administration of immunosuppressive medications is inevitably associated with increased risk of infection and malignancy due to the sustained suppression of anti-microbial and anti-tumor immunity [3]. Therefore, new therapeutic agents, which could suppress detrimental immune response without causing life-threatening immunosuppression are urgently needed for the treatment of autoimmune and inflammatory diseases.

Due to their capacity to modulate phenotype and function of immune cells, mesenchymal stem cells (MSCs) have been considered as potentially new remedy for the treatment of autoimmune and inflammatory diseases [4]. Although the large number of experimental and clinical studies demonstrated beneficial effects of MSCs in alleviation of immune cell-driven, organ-specific and systemic inflammatory disorders [4], several safety concerns related to the MSC-based therapy has been raised [5]. Unwanted differentiation of transplanted MSCs and their possible malignant transformation have been identified as the most important safety issues [5]. Additionally, patients suffering from inflammatory bowel diseases (IBDs) and idiopathic pulmonary fibrosis (IPF) who received immunosuppressive drugs just before MSC injection developed severe respiratory and gastrointestinal infections, indicating that MSCs should not be used immediately after or in combination with other immunosuppressive agents [6,7,8]. Autologous transplantation of MSCs is difficult to attempt on patients with fulminant diseases because of a long cell preparatory period and cell transplantation timing. Therefore, allogeneic MSCs have been used for the attenuation of acute and fulminant inflammation [4]. However, MSCs express major histocompatibility complex (MHC) class I molecules and may elicit strong allogeneic immune responses in MHC-class I-mismatched recipients that could result in life-threatening aggravation of on-going inflammation [9]. 

A large number of experimental and clinical studies revealed that most of MSC-based immunomodulatory effects were attributed to the immunoregulatory properties of MSC-sourced secretome, which consists of a soluble component and encapsulated extracellular vesicles (MSC-EVs): apoptotic bodies, microvesicles and exosomes (MSC-Exos) [10]. Subcellular particles derived from dead or dying MSCs might contribute to the therapeutic efficacy of transplanted MSCs [11]. Accordingly, during the apoptotic loss of MSCs, MSC-derived immunoregulatory factors are within large EVs (apoptotic bodies with diameter >1000 nm) delivered to the phagocytes, inducing alteration in their phenotype and function [11,12,13]. Microvesicles (100–1000 nm) and Exos (30–200 nm) are nano-sized MSC-sourced EVs, which are, after the budding from the plasma membrane, released into the extracellular milieu where it exerts biological effects in a paracrine and endocrine manner [14]. 

MSC-derived EVs express several adhesion molecules (CD29, CD44 and CD73), which enable their homing to the injured and inflamed tissues. In the mouse model of acute kidney injury (AKI), MSC-EVs were mainly accumulated in the inflamed kidneys [15], while in an intracerebral hemorrhage model, MSC-EVs were detected in the injured brains [12]. Nevertheless, most of intravenously injected MSC-EVs accumulate in the liver, spleen and the lungs where the mononuclear phagocyte system (MPS) is active [12]. Clearance of EVs from the circulation was much slower in macrophage-depleted mice, indicating the important role of MPS in EVs biodistribution. Therefore, several research groups used a membrane-editing technology to induce surface modifications of MSC-EVs in order to increase their chances for reaching the target cells before being taken up by the MPS. Alvarez and colleagues increased neurotropism of EVs by modifying their membrane protein Lamp2B with Rabies viral glycoprotein (RVG), which specifically binds to the acetylcholine receptors on neuronal cells. Accordingly, EVs displaying the RVG protein more specifically targeted neuronal cells and showed better therapeutic effects in attenuation of Alzheimer’s disease than naïve EVs [16]. Kooijman and coworkers used glycosylphosphatidylinositol (GPI), a glycolipid, which is integrated into the EV’s membrane during their biogenesis, to incorporate chemokine receptors, enzymes, antibodies and signaling molecules on EV’s membranes, enhancing their tropism and therapeutic potential [17].

MSC-EV’s membrane is enriched in cholesterol, sphingomyelin, ceramide and lipid raft proteins [9] enabling membrane fusion with target cells and trafficking of MSC-EVs through the body, regardless of biological barriers [10]. It was suggested that MSC-EVs, in a similar manner as tumor-derived EVs, crossed the blood–brain barrier (BBB) and that transcytosis is the main underlying mechanism [18]. Endothelial recycling endocytic pathway is involved in the transcellular transport of EVs [19]. Transcytosis through the brain endothelial cells includes clathrin-dependent and caveolin-independent endocytosis of EVs, their intracellular trafficking (driven by Rab11-expressing recycling endosomes) and vesicle associated membrane protein (VAMP)-3/Snap23/syntaxin 4-dependent release of EVs into the extracellular environment [20]. 

Upon reaching their target cells, MSC-EVs may trigger signaling via the receptor–ligand interaction, or be internalized by endocytosis to deliver their content [21]. All MSC-EVs are enriched with MSC-sourced bioactive molecules (messenger RNA (mRNA) and microRNAs (miRNAs)), enzymes, cytokines, chemokines, immunomodulatory and growth factors) that regulate phenotype, function, survival and homing of immune cells [14]. Accordingly, immunosuppressive effects elicited by MSC-EVs were similar to those observed after transplantation of MSCs [10]. As a cell-free product, MSC-derived EVs overcomes all safety concerns related to the long-term survival of engrafted MSCs, including their un-controlled differentiation, malignant alteration or rejection due to the activation of allogeneic immune response in MHC-mismatched recipients [14]. Importantly, composition of MSC-EVs can be modulated by MSCs’ preconditioning in vitro, enabling generation of disease-specific, MSC-based, immunosuppressive product, which could be used as a new remedy in cell-free treatment of autoimmune and inflammatory diseases [14].

In this review article we emphasized current knowledge regarding molecular and cellular mechanisms responsible for the therapeutic effects of MSC-EVs in attenuation of autoimmune and inflammatory diseases. We described the disease-specific cellular targets of MSC-EVs and defined MSC-sourced molecules, which were mainly responsible for MSC-EV-based protection of injured cells and/or immunosuppression. An extensive literature review was carried out in October 2019 across several databases (MEDLINE, EMBASE, Google Scholar, ClinicalTrials.gov), from 1991 to present. Keywords used in the selection were: “mesenchymal stem cells (MSCs)”, “extracellular vesicles (EVs)”, “exosomes (Exos)”, “inflammation”, “macrophages”, “dendritic cells”, “neutrophils”, “T cells”, “gut epithelial cells”, “hepatocytes”, “hepatic stellate cells”, “lung epithelial cells”, “renal tubular cells”, “retinal cells”, “neurons” and “cardiomyocytes”. All journals were considered, and an initial search retrieved 1837 articles. The abstracts of all these articles were subsequently reviewed by three of the authors (CRH, VD and VV) to check their relevance to the subject of this manuscript. Eligible studies had to delineate molecular and cellular mechanisms involved in the beneficial effects of MSC-derived EVs and their findings were analyzed in this review.

## 2. Macrophages: The Main Cellular Targets of MSC-Derived EVs in Alleviation of Colon Inflammation

Macrophages have been identified as the most important cells for the induction of colon inflammation [22,23]. Massive release of damage-associated molecular patterns (DAMPs) from injured epithelial cells activates NF-κB signaling pathway in colon macrophages, resulting in increased expression of inducible nitric oxide synthase (iNOS) and enhanced secretion of inflammatory cytokines (tumor necrosis factor alpha (TNF-α), IL-1β), nitric oxide (NO) and lymphocyte and monocyte-recruiting chemokines (CCL-17 and CCL-24) [24]. Macrophage-derived TNF-α and IL-1β induce enhanced expression of E and P selectins on endothelial cells enabling massive influx of circulating monocytes and lymphocytes in the injured gut [22]. Macrophage-sourced CCL-17 and CCL-24 attract inflammatory M1 macrophages and IFN-γ producing CD4+Th1 cells, which either directly damage epithelial cells (NO-producing M1 macrophages) or activate macrophages in IFN-γ-dependent manner (Th1 cells) and indirectly promote colon injury and inflammation by enabling creation of “positive inflammatory loop” in the gut [22]. 

**Figure 1:**
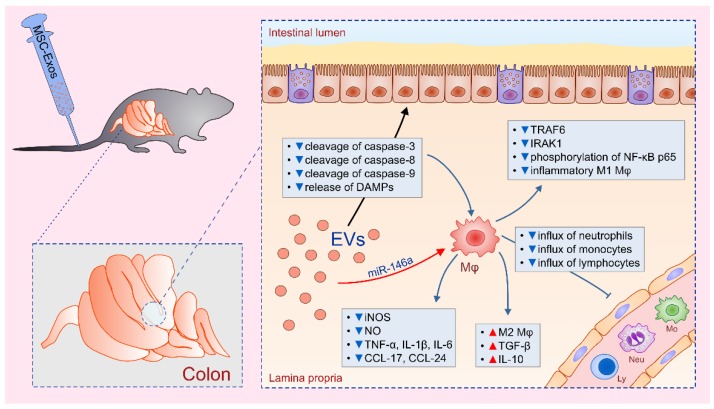
Modulation of phenotype and function of colonic macrophages as the main mechanism for mesenchymal stem cell-derived extracellular vesicle (MSC-EV)-based attenuation of ulcerative colitis: MSC-EVs reduced cleavage of caspase-3, -8 and -9 and alleviated release of damage-associated molecular patterns (DAMPs) from injured gut epithelial cells, resulting in attenuated activation of NF-κB signaling pathway in colon macrophages. Through the delivery of miR-146a, MSC-EVs inhibited TNF receptor-associated factor 6 (TRAF6) and IL-1 receptor-associated kinase 1 (IRAK1) expression, down-regulated phosphorylation of NF-κB p65 and inhibited generation of inflammatory M1 phenotype in macrophages, which was manifested by down-regulated expression of inducible nitric oxide synthase (iNOS), significantly reduced production of nitric oxide (NO), inflammatory cytokines (TNF-α, IL-1β, IL-6) and chemokines (CCL-17 and CCL-24) and resulted in reduced influx of circulating neutrophils, monocytes and lymphocytes in the inflamed gut. Additionally, MSC-EVs induced polarization of colon macrophages in anti-inflammatory M2 phenotype, manifested by increased secretion of immunosuppressive cytokines TGF-β and IL-10 and alleviation of colitis.

Several recently published studies indicated that MSC-based alleviation of colitis was mainly relied on MSC-EV-induced suppression of colon macrophages [25,26,27,28] (Figure 1). Cao and colleagues showed that MSC-EVs significantly alleviated dextran sulphate sodium (DSS)-induced colitis in mice by inducing polarization of colon macrophages in immunosuppressive, M2 phenotype [25]. Higher number of IL-10-producing M2 macrophages, observed in MSC-EVs-treated mice, correlated with reduced weight loss, alleviated injury of gut epithelial cells and increased colon length [25]. Concentration of macrophage-sourced inflammatory cytokines and chemokines (TNF-α, CCL-17 and CCL-24) and Th1-derived IFN-γ were significantly attenuated in the gut of DSS-treated mice that received MSC-EVs. Additionally, MSC-EVs managed to increase colon concentration of immunosuppressive cytokines (IL-10 and transforming growth factor beta (TGF-β)), enabling enhanced repair and regeneration of DSS-injured epithelial cells [25]. Importantly, in vitro obtained results confirmed that MSC-EVs entered in lipopolysaccharides (LPS)-activated colon macrophages, suppressed production of inflammatory cytokines and induced generation of immunosuppressive M2 phenotype [25]. 

The main mechanism responsible for MSC-EV-induced inhibition of colon macrophages relies on the suppression of NF-κB and iNOS-driven signaling [26,27]. Administration of MSCs-EVs down-regulated expression of NF-κB p65 and reduced production of NO, IL-1β and IL-18 in colon macrophages, resulting in alleviated 2,4,6-trinitrobenzene sulfonic acid (TNBS)-induced colitis [26,27]. Wu and coworkers suggested that microRNA-146a (miR-146a), a well-known anti-inflammatory miRNA, acted as a negative feedback regulator of colon macrophages in MSC-EV-based alleviation of gut inflammation [27]. Administration of EVs, obtained from miR-146-overexpressing MSCs, inhibited TNF receptor-associated factor 6 (TRAF6) and IL-1 receptor-associated kinase 1 (IRAK1) expression, down-regulated phosphorylation of NF-κB p65 and inhibited generation of inflammatory phenotype in macrophages, attenuated production of TNF-α, IL-1β, IL-6 and reduced colon injury and inflammation [27]. 

Yang and colleagues suggested that modulation of anti-oxidant/oxidant balance in the injured gut was responsible for MSC-EVs-induced effects on macrophage phenotype and function [26]. MSC-EV-mediated suppression of NO-driven injury in the gut was accompanied by decreased activity of myeloperoxidase and malondialdehyde and increased superoxide dismutase and glutathione activity. Furthermore, MSC-EVs reduced cleavage of caspase-3, -8 and -9 and alleviated release of DAMPs from injured gut epithelial cells, resulting in attenuated activation of NF-κB signaling pathway in colon macrophages, which consequently led to the generation of immunosuppressive M2 phenotype [26].

Mao and coworkers suggested that, in addition to the inhibition of NF-κB and iNOS, MSC-EVs exert their beneficial effects in colitis through the inhibition of IL-7 signaling in colon macrophages, as well [28]. IL-7 displays strong chemotactic property for circulating monocytes enabling their massive accumulation in inflamed tissues [29]. Additionally, IL-7 induces increased production of NO, TNF-α and IL-1β in macrophages, enhancing their inflammatory properties [29]. MSC-EVs contain miR17, which impairs IL-7:IL-7 receptor signaling by preventing synthesis and transactivation of Janus kinase 1 [30,31]. In line with these findings, MSC-EVs treatment significantly reduced activation of IL-7 and iNOS-signaling pathways in colon macrophages, resulting in attenuated production of TNF-α, IL-1β, IL-6 and increased secretion of IL-10, which led to the alleviation of colitis [28]. 

## 3. Molecular Mechanisms Responsible for MSC-EVs-Based Protection of Hepatocytes in Acute Liver Injury and Fibrosis

As recently evidenced by us and others, MSC-derived secretome efficiently attenuated acute liver failure and liver fibrosis in mice by suppressing major effector cells: natural killer T cells (NKT) cells in fulminant hepatitis and CD4+ T helper lymphocytes and hepatic stellate cells (HSCs) in fibrosis [32,33,34,35,36].

MSC-sourced secretome contains high concentration of NO and reactive nitrogen species, which decrease proliferation of liver NKT cells [37]. Accordingly, administration of MSC-derived secretome significantly reduced total number of inflammatory NKT cells in the injured livers of mice with fulminant hepatitis [32,34]. Additionally, MSC-derived secretome contains Kynurenine, which maintains immunosuppressive phenotype of FoxP3-expressing NKT cells in the inflamed livers and suppress their transdifferentiation in inflammatory, IL-17-producing NKT17 cells [34]. Liver NKT cells cultured in the presence of MSC-sourced secretome have reduced capacity for production of hepatotoxic (TNF-α) and inflammatory cytokines (IFN-γ, IL-17) [32,34]. Moreover, reduced expression of molecules, which are responsible for NKT cell-dependent apoptosis of hepatocytes (Fas ligand, CD107a and NKG2D) was observed in liver NKT cells cultured in the presence of MSC-derived secretome [34].

In addition to immunosuppressive effects against NKT cells, MSC-sourced secretome may directly protect hepatocytes from cell death [38,39]. Injection of human menstrual blood-derived MSC-Exos significantly attenuated d-galactosamine/lipopolysaccharide (d-GalN/LPS)-induced acute liver injury and increased survival rate of experimental mice by suppressing caspase-3-driven apoptosis of hepatocytes [38]. In line with these findings are results obtained by Chen and colleagues who provided additional evidence of anti-apoptotic capacity of MSC-EVs in a murine model of autoimmune hepatitis (AIH) [39]. Hepatoprotective effects of MSC-Exos were relied on suppression of NLRP3-dependent activation of caspase-1 and on inhibition of caspase-1-driven pyroptosis, characterized by plasma membrane rupture, cytoplasmic swelling, osmotic lysis, DNA cleavage and massive release of pro-inflammatory cytokines (IL-1β and IL-18) [40]. Accordingly, by suppressing pyroptosis, MSC-Exos inhibited cell death of hepatocytes and attenuated IL-1β and IL-18-driven inflammation. MSC-derived miR-233 was crucially important for these hepatoprotective effects of MSC-Exos since administration of Exos derived from miR-233 deficient MSCs did not attenuate AIH [39]. The analysis of NLRP3-signaling pathway revealed that exosomal miR-233 suppressed NLRP3:caspase-1-induced pyroptosis by inducing degradation of NLRP3 mRNA in hepatocytes [39]. MSC-Exos attenuate oxidative stress in inflamed livers, as well [41]. MSC-Exo-derived glutathione peroxidase 1 (GPX1) was mainly responsible for MSC-Exo-dependent suppression of reactive oxygen species (ROS) formation in injured hepatocytes [41].

In addition to their hepatoprotective effects, MSC-Exos may induce proliferation of hepatocytes. As recently evidenced by Du and colleagues intravenous injection of Exos, obtained from human-induced pluripotent stem cell-derived MSCs (hiPSC-MSCs-Exos) attenuated hepatic ischemia-reperfusion (I/R) injury by suppressing necrosis of hepatocytes and by promoting their proliferation [42]. The serum levels of hepatocyte injury markers (aspartate aminotransferase (AST) and alanine aminotransferase (ALT) were significantly lower and the expression levels of proliferation markers (proliferation cell nuclear antigen (PCNA) and phosphohistone-H3 (PHH3)) were greatly increased in the livers of I/R-injured mice that received hiPSC-MSCs-Exos [42]. Significantly increased proliferation of hiPSC-MSCs-Exos-treated primary hepatocytes and HL7702 human hepatocytes was confirmed in vitro. Mechanistically, hiPSC-MSCs-Exos directly fused with target hepatocytes or HL7702 cells and increased the activity of sphingosine kinase (SK1) resulting in synthesis of sphingosine-1-phosphate (S1P), which promoted hepatocyte growth, survival and proliferation [42,43]. This phenomenon was completely abrogated after inhibition of either SK1 or S1P receptor, confirming crucial importance of SK1/S1P signaling for hiPSC-MSCs-Exos-induced enhanced proliferation of hepatocytes [42].

Several lines of evidence demonstrated that MSC-EVs protected hepatocytes during chronic liver inflammation and fibrosis, as well [44]. Results obtained by Li and colleagues showed that human umbilical cord-MSCs-derived Exos attenuated carbon tetrachloride (CCl4)-induced liver fibrosis in mice, as evidenced by recovered serum AST levels and reduced deposition of collagen type I and III in the liver [44]. Significantly decreased expression of TGF-β1 and phosphorylated Smad2 was observed in the CCl4-injured livers of MSC-Exo-treated mice, indicated that MSC-Exo-dependent inhibition of TGF-β1 signaling pathway in hepatocytes was crucially important for anti-fibrotic effects of MSC-Exos. Upon phosphorylation, Smad2 formed complexes with phosphorylated Smad3 and Smad4 and, subsequently, translocated into the nucleus to regulate the transcription of genes responsible for epithelial-to-mesenchymal transition (EMT) of hepatocytes [45]. Significant increase in E-cadherin-positive cells and decrease in N-cadherin- and vimentin-positive cells in MSC-Exo-treated fibrotic livers, suggested that MSC-Exos prevented TGF-β1/Smad2-induced EMT of hepatocytes [44]. This hypothesis was confirmed in vitro. MSC-Exos completely reversed spindle-shaped morphology and abrogated expression of EMT-associated markers in HL7702 human hepatocytes that underwent EMT after treatment with recombinant TGF-β1 [44].

MSC-EVs attenuated chronic liver inflammation by suppressing production of inflammatory cytokines (TNF-α, IL-1β and IL-6) and pro-fibrotic TGF-β1in liver macrophages (Kupffer cells), while HSCs were the main cellular targets in MSC-EVs-based alleviation of liver fibrosis [46]. Through the production of inflammatory cytokines and monocyte and lymphocyte-attracting chemokines, Kupffer cells attract circulating leucocytes in inflamed liver contributing to the progression of inflammation [47]. Furthermore, through the production of TGF-β1, Kupffer cells induce enhanced expression of pro-fibrotic genes (collagen I, vimentin, α-SMA and fibronectin) in HSCs, resulting in the development of liver fibrosis [47]. In line with these findings, Qu and coworkers engineered miRNA-181-5p-overexpressing adipose tissue derived MSCs (MSCs^miRNA-181-5p^), which produced Exos that efficiently alleviated liver fibrosis by affecting survival and pro-fibrotic function of HSCs [48]. MSCs^miRNA-181-5p^-Exos promoted expression of autophagy-related Beclin-1 and inhibited expression of anti-apoptotic Bcl-2 in HSCs, resulting in increased apoptosis and autophagy of HSCs in fibrotic livers. Furthermore, MSCs^miRNA-181-5p^-Exos significantly down-regulated expression of pro-fibrotic genes (collagen I, vimentin, α-SMA and fibronectin) in HSCs, which led to the attenuation of CCl4-induced liver fibrosis in MSCs^miRNA-181-5p^-Exos-treated mice [48].

## 4. MSC-EVs as Next-Generation Therapeutics for the Treatment of Lung Inflammatory Diseases

There is growing evidence that MSC-EVs protect lung epithelial cells from reactive oxidative species and proteolytic enzymes released by lung-infiltrating neutrophils and monocytes [49,50,51,52]. Li and colleagues demonstrated that MSC-EV-based protection of lung epithelial cells against oxidative stress-induced cell death is dependent on anti-apoptotic properties of miR-21-5p [50]. Intratracheal administration of MSC-Exos inhibited both intrinsic and extrinsic apoptotic pathways in lung epithelial cells. However, pre-treatment of MSCs with miR-21-5p antagomir completely abrogated MSC-Exos-mediated suppression of caspase-3, -8 and -9 and diminished MSC-Exo-based protective effects [50]. Western blot analysis revealed that pro-apoptotic phosphatase and tensin homolog (PTEN) and programmed cell death protein 4 (PDCD4) were the main targets of MSC-derived miR-21-5p since their expression was significantly decreased in lung epithelial cells of MSC-Exo-treated mice. When I/R-injured mice received Exos derived from miR-21-5p-antagomir-treated MSCs, expression of PTEC and PDCD4 and apoptosis of lung epithelial cells were not reduced, indicating crucial importance of miR-21-5p-dependent suppression of PTEN and PDCD4 for anti-apoptotic effects of MSC-Exos in I/R lung injury [50].

In addition to their anti-oxidative effects, MSC-EVs may protect lung epithelial cells by regulating protease/antiprotease balance in the inflamed lungs [51]. Alpha-1-antitrypsin (AAT) is a potent inhibitor of neutrophil-derived proteolytic enzymes, which protects lung epithelial cells and exerts important anti-inflammatory and immunomodulatory effects in the lungs [52]. Most recently, Bari and colleagues revealed that AAT was aggregated and/or adsorbed on the surface of adipose-tissue derived MSC-EVs that served as natural carriers of AAT, promoting its stability and activity in vivo [51]. Importantly, MSC-EVs derived from IL-β-primed MSCs showed significantly higher expression of AAT gene and had increased anti-elastase activity compared to MSC-EVs obtained from IL-β-non-primed MSCs [51]. 

Importantly, MSC-EVs, in addition to AAT, contained 46 proteins involved in the response to Gram-negative bacteria, implying potent anti-microbial activity of MSC-EVs [51]. In line with these findings are results obtained by Hao and colleagues who demonstrated that administration of MSC-EVs remarkably reduced severity of bacterial pneumonia in mice [53]. MSC-EVs increased phagocytic and anti-microbial activity of lung-infiltrating neutrophils and monocytes by promoting synthesis of leukotriene B4 (LTB4) [53]. LTB4 is well-known activator of leucocytes, which augments phagocytosis and promotes release of anti-microbial agents, contributing to the bacterial clearance [54]. Hao and colleagues demonstrated that miR-145, contained within MSC-EVs, reduced expression of multidrug resistance-associated protein 1 (MRP1) in lung macrophages [53]. MRP1 is ATP-binding cassette transporter, which inhibits synthesis and release of LTB4 [53]. Accordingly, MSC-EV-induced suppression of MRP1 resulted in enhanced release of LTB4 by alveolar macrophages that, due to its anti-microbial activity, increased bacterial clearance and reduced severity of bacterial pneumonia in mice [53].

It is important to highlight that capacity of MSC-EVs to modulate phenotype and function of alveolar macrophages depends on the phase of anti-microbial inflammatory response [55]. During the onset of inflammation, MSC-EVs, in a miR-145/LTB4-dependent manner, promote phagocytic activity of alveolar macrophages contributing to the elimination of bacterial pathogens from the lungs. However, during the resolution of inflammation, MSC-EVs promote expansion of alternatively activated M2 macrophages that are involved in tissue repair and regeneration [55]. It is well known that alveolar macrophages, through the production of inflammatory cytokines and chemokines, orchestrate influx of circulating monocytes and lymphocytes in inflamed lungs, promoting chronic inflammation [55]. Therefore, MSC-EV-based suppression of chronic, macrophage-driven inflammatory lung diseases was mainly relied on MSC-EV-dependent polarization of alveolar macrophages. MSC-Exos significantly decreased iNOS mRNA expression and remarkable increased expression of Arginase-1 mRNA in alveolar macrophages, inducing their polarization from inflammatory M1 towards immunosuppressive M2 phenotype [50,55]. Accordingly, concentration of M1-related inflammatory cytokines (IL-8, IL-1β, IL-6 and TNF-α) was significantly reduced and concentration of M2 macrophage-derived immunosuppressive cytokines (IL-10 and TGF-β) was increased in the lungs of I/R-injured mice that received MSC-Exos [50]. 

Interestingly, as recently revealed by Huang and colleagues, aging MSC-EVs did not manage to induce generation of M2 macrophages in the inflamed lungs [56]. Although aging and young MSC-EVs had similar phenotypic characteristics (expression of CD63, CD81, CD105 and CD44), their capacity to alter the phenotype of alveolar macrophages was different. Internalization of aging MSC-EVs by alveolar macrophages was significantly lower compared to the young MSC-EVs. Furthermore, aging MSC-EVs had reduced capacity to inhibit production of inflammatory, M1-related cytokines (IL-6, IL-1β and TNF-α) and to induce expression of M2-related Arginase-1 in alveolar macrophages [56]. Most importantly, aging and young MSC-EVs differed in levels of miRNAs (miR-223-5p, miR-127-3p and miR-125b-5p) that regulate macrophage polarization. Compared with aging MSC-EVs, young MSC-EVs showed higher expression of miR-223-5p (which is responsible for induction of M2 phenotype in alveolar macrophages) and lower expression of miR-127-3p and miR-125b-5p (which promote generation of M1 phenotype in macrophages) [56]. Since aging MSC-Exos had significantly reduced capacity to attenuate M1 macrophage driven inflammation in the lungs, MSC-Exos used for the therapy of inflammatory lung diseases should be obtained only from young donors.

Mansouri and colleagues recently revealed that single intravenous administration of Exos, obtained from human bone marrow-derived MSC, managed to significantly attenuate bleomycin-induced lung fibrosis in mice through the modulation of phenotype and function of alveolar macrophages [57]. An improved Ashcroft score and reduced deposition of collagen were observed in bleomycin-injured lungs of MSC-Exo-treated animals. MSC-Exo-based alleviation of fibrosis was followed by significantly reduced number of TGF-β1-producing, Arginase-1 and CD206-expressing alveolar macrophages, indicating that macrophages were the main cellular targets of MSC-Exos in alleviation of pulmonary fibrosis. Importantly, anti-fibrotic effects were not observed in bleomycin-injured mice that received fibroblasts-derived Exos or Exos free iodixanol, suggesting that immunomodulatory properties of MSCs were responsible for beneficial effects of MSC-Exos [57].

In addition to alveolar macrophages, MSC-EVs may also modulate phenotype and function of lung-infiltrating dendritic cells (DCs) [58]. As recently evidenced by Cho and colleagues, MSC-EV-based alleviation of Th2 cell-driven immune response against *Aspergillus* protease antigen was dependent on suppression of antigen-presenting properties of DCs [45]. MSC-Exos induced increased expression of immunosuppressive IL-10 and TGF-β that suppressed maturation of lung DCs [58]. Immature DCs of MSC-Exos-treated mice had reduced expression of co-stimulatory molecules (CD40, CD80 and CD86) and were not capable to optimally activate CD4+Th2 cells, resulting in alleviation of Th2 cell-driven lung inflammation [58].

The lung is a portal of entry for numerous microbial pathogens, which are, immediately after invasion, captured and efficiently eliminated by alveolar macrophages and lung DCs, resulting in the activation of antigen specific, T cell-driven immune response [59,60]. Upon activation, alveolar macrophages and lung DCs produce large amount of inflammatory chemokines and cytokines and orchestrate both local and systemic immune response [59]. Accordingly, lung macrophages and DCs have been considered as the cells that are crucially important for the generation and development of chronic inflammatory diseases [59]. Since most of intratracheally and intravenously administered MSC-EVs accumulate in the lungs where, in similar manner as microbial pathogens, become phagocyted by lung-infiltrated macrophages and DCs, capacity of MSC-EVs to modulate phenotype and function of these professional antigen-presenting cells could be used not only for alleviation of inflammatory lung diseases but also for modulation of detrimental macrophage and DC-driven systemic immune response.

## 5. Modulation of Microglial Activity: The Main Mechanism Responsible for MSC-EVs-Dependent Attenuation of Neuroinflammatory Diseases

Microglia, the resident immune cells of the central nervous system (CNS), maintain tissue homeostasis under physiological conditions [61]. However, after neuronal injury, microglia secrete pro-inflammatory cytokines that either have direct neurotoxic effects or, in combination with inflammatory chemokines, promote influx of circulating neutrophils in inflamed tissue [61]. An excessive microglial activation damages the surrounding healthy neural tissue and induces the release of alarmins and DAMPs from dead or dying neurons, which in turn, activates microglia enabling creation of “positive inflammatory loop” in CNS, that results in a massive and progressive loss of neurons [61]. In line with these findings, Ding and colleagues recently revealed that modulation of microglial activity was the main mechanism responsible for beneficial effects of MSC-EVs in alleviation of Alzheimer’s disease (AD) [62]. Excessive accumulation of the amyloid-β peptide (Aβ) in the brain is considered as the most common pathological characteristic of AD, which triggers dysfunction of cognitive behavior [63]. Intravenously injected Exos, obtained from human umbilical cord-derived MSCs, managed to reduce Aβ deposition and increased spatial learning and memory function in AβPP/PS1 transgenic mice, used as murine model of AD [62]. Additionally, Bodart-Santos and colleagues recently revealed that MSC-EVs prevented neuronal damage in AD by suppressing oxidative stress-induced injury of hippocampal neurons [64]. Catalase was mainly responsible for MSC-EV-based protection against ROS-induced injury since MSC-EVs with inactivated catalase were unable to prevent ROS formation in hippocampal neurons [64]. MSC-Exos induced polarization of microglia towards immunosuppressive M2 phenotype. Significantly higher number chitinase 3-like 3, arginase-1 and mannose receptor C type 1 (MRC1)-expressing M2 microglia cells were found in the brains of MSC-Exos-treated AβPP/PS1 mice [62]. M2 cells produce Aβ-degrading enzymes (neprilysin (NEP) and insulin-degrading enzyme (IDE)) and anti-inflammatory cytokines (IL-10 and TGF-β), contributing to the reduced Aβ deposition and alleviated inflammation [61]. Significantly increased levels of NEP, IDE, IL-10 and TGF-β, and greatly reduced concentration of inflammatory cytokines (TNF-α and IL-1β) were noticed in the brains of MSC-Exos-treated AβPP/PS1 mice, indicating that MSC-Exos induce conversion of microglia from inflammatory M1 towards immunosuppressive M2 phenotype [62]. MSC-Exo-induced alternative microglial activation was confirmed in vitro, since significantly higher concentration of IL-10 and TGF-β and lower concentration of TNF-α and IL-1β were measured in supernatants of MSC-Exo-treated BV2 murine microglia cells [62].

Modulation of microglial activity was mainly responsible for beneficial effects of MSC-Exos in alleviation of multiple sclerosis (MS), inflammation-mediated demyelinating disease [65]. Significantly improved motor function was noticed in Theiler’s murine encephalomyelitis virus (TMEV)-infected mice that received MSC-EVs [65]. Remarkably reduced number of Iba-1-positive microglia cells was observed in the brains of TMEV+MSC-EV-treated mice compared to TMEV-only treated animals [65]. Importantly, MSC-EVs altered cytokine milieu in TMEV-infected mice. Significantly lower concentration of microglia-derived inflammatory cytokines (TNF-α, IL-1-β, IL-18, IL-6 and IL-12) was noticed in TMEV+MSC-EV-treated mice [65]. Furthermore, MSC-EVs significantly alleviated concentration of Th1 cell-derived IFN-γ and Th17 cell-sourced IL-17A, indicating that, in addition to microglia, MSC-EVs suppressed inflammatory properties of brain-infiltrating inflammatory CD4+T cells, as well [65].

As recently revealed by Shiue and colleagues [66], continuous intrathecal injection of MSC-Exos enabled functional recovery from nerve ligation-induced injury [66]. MSC-Exos suppressed production of inflammatory cytokines (TNF-α and IL-1β) and promoted synthesis of anti-inflammatory cytokines (IL-10 and TGF-β) in microglia, resulting in the alleviation of inflammation within the site of neural injury [66]. The analgesic effects of MSC-Exos involved their actions on neurons, as well. MSC-Exos delivered brain-derived neurotrophic factor and glial cell line-derived neurotrophic factor in the ipsilateral L5/6 dorsal root ganglion of nerve-ligated rats, enabling better recovery from nerve ligation-induced injury [66]. Protein analysis demonstrated that vascular endothelial growth factor C, angiopoietin-2 and fibroblast growth factor-2 were also present in the MSC-Exos, indicating that induction of neo-angiogenesis may be, at least partially responsible for beneficial effects of MSC-Exos. Importantly, immunofluorescence staining showed that MSC-Exos were presented in the ipsilateral L5 spinal dorsal horn, dorsal root ganglion and peripheral axons, suggesting a high homing ability of MSC-Exos [66].

Huang and colleagues provided evidence that MSC-Exos ameliorated cerebral I/R injury by preventing neural cell death through the inhibition of caspase-9 and caspase-3 [67]. MSC-sourced pigment epithelium-derived factor (PEDF), which exhibits anti-inflammatory, antioxidative and neuroprotective properties, was mainly responsible for beneficial effects of MSC-Exos [68]. Through the delivery of PEDF, MSC-Exos increased expression of autophagy-associated protein LC3 and suppressed caspase-3-driven apoptosis in neurons, significantly reducing I/R-induced injury [67]. Exos, obtained from PEDF-overexpressing MSCs showed better therapeutic effects, while inhibition of autophagy significantly reduced neuroprotection elicited by PEDF-containing MSC-Exos, indicating crucial importance of PEDF-induced autophagy for MSC-Exo-based attenuation of cerebral I/R injury [67].

## 6. Molecular Mechanisms Responsible for MSC-EVs-Based Renal Protection

**Figure 2:**
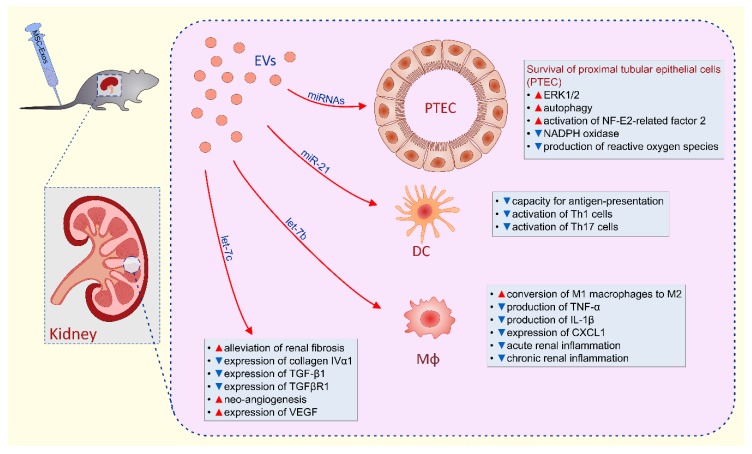
Molecular mechanisms responsible for MSC-EVs-based renal protection: MSC-EVs-dependent renal protection during acute kidney injury (AKI) is relied on inhibition of apoptosis, necrosis and oxidative stress and the promotion of autophagy in renal tubular epithelial cells as well as suppression of detrimental immune response. Through the delivery of messenger RNAs (mRNAs), MSC-EVs induce enhanced expression of ERK1/2 and promote survival of proximal tubular epithelial cells (PTEC). MSC-EVs activated autophagy in PTEC and protected against cisplatin-induced AKI by delivering trophic factor 14-3-3ζ, which interacted with ATG-16L, a protein essential for autophagy induction. MSC-EVs enhanced activation of NF-E2-related factor 2/antioxidant responsive element, decreased expression of NADPH oxidase and reduced production of reactive oxygen species in ischemic kidneys and promoted their regeneration. Additionally, through the delivery of miR-21, MSC-EVs significantly attenuated capacity for antigen-presentation of renal dendritic cells, which resulted in reduced activation of Th1 and Th17 cells and alleviation of Th1 and Th17 cell-driven inflammation in the kidneys. Through the delivery of microRNAs (miRNAs), particularly let-7b, MSC-EVs induced conversion of inflammatory M1 macrophages into immunosuppressive M2 cells, which produced lower amount of inflammatory cytokines (TNF-α and IL-1β) and chemokine CXCL1, resulting in alleviated acute and chronic renal inflammation. MSC-sourced miRNA, particularly let-7c, targeted pro-fibrotic genes (collagen IVα1, TGF-β1 and TGFβR1) in inflamed kidneys, crucially contributing to the therapeutic effects of MSC-EVs in renal fibrosis. Additionally, neo-angiogenesis, induced by MSC-derived vascular endothelial growth factor (VEGF) was also responsible for beneficial effects of MSC-EVs in alleviation of renal fibrosis.

MSC-EVs-dependent renal protection is relied on the inhibition of apoptosis, necrosis and oxidative stress in renal tubular epithelial cells as well as suppression of detrimental immune response in the kidneys (Figure 2) [69]. MSC-sourced mRNAs, miRNAs and immunosuppressive factors were mainly responsible for beneficial effects of MSC-EVs in alleviation of acute and chronic renal inflammation [69,70,71,72,73,74,75,76,77,78,79,80].

Several lines of evidence demonstrated that MSC-derived mRNAs were involved in MSC-EVs-based attenuation of acute kidney injury (AKI) [70,71,72,73]. Bruno and colleagues noticed significantly improved renal function in glycerol and cisplatin-injured kidneys of experimental animals [70,71,72]. They revealed that mRNAs, which regulate transcription (e.g., CLOCK, IRF6 and LHX6), cell cycle regulation (e.g., SENP2, RBL1 and CDC14B) and DNA/RNA repair (e.g., HMGN4, TOPORS and ESF1) were contained within MSC-EVs and suggested that these MSC-derived mRNAs were mainly responsible for increased proliferation and suppressed apoptosis of renal tubular cells in cisplatin + MSC-EV-treated animals [70,71,72]. Pretreatment with ribonucleases (RNase) completely abolished MSC-EVs-based renoprotection, confirming that MSC-derived mRNAs were crucially involved in MSC-EV-dependent alleviation of AKI [70,71,72]. The same conclusion was made by Ju and coworkers who observed that RNase treatment abolished MSC-EVs-induced overexpression of ERK1/2 in renal tubular epithelial cells and completely abrogated therapeutic effects of MSC-EVs in I/R-induced AKI [73].

In line with these findings are results reported by Gatti and colleagues who demonstrated that MSC-EVs alleviated I/R-induced AKI by reducing apoptosis and by increasing proliferation of renal tubular cells [74]. Similarly as it was observed by Bruno et al. [70] and Ju and et al [73], MSC-EV-based renoprotection was diminished by RNase pretreatment [74], confirming the hypothesis that beneficial effects of MSCs-EVs were mainly mediated by MSC-sourced mRNA.

Wang and colleagues indicated that activation of autophagy in proximal tubular epithelial cells (PTEC) was responsible for greatly improved renal function of cisplatin + MSC-EVs-treated mice [75]. They showed that beneficial effects of MSC-EVs were completely abrogated by autophagy inhibitor, 3-methyladenine. Similarly, Jia and coworkers demonstrated that MSC-EVs activated autophagy in cisplatin-injured PTEC and protected against AKI by delivering trophic factor 14-3-3ζ, which interacted with ATG-16L, a protein essential for autophagy induction [76].

By using the I/R model of AKI, Zhou and colleagues indicated that attenuation of oxidative stress was mainly responsible for MSC-EVs-based renoprotection in AKI [77,78]. This hypothesis was based on enhanced activation of NF-E2-related factor 2/antioxidant responsive element, decreased expression of NADPH oxidase and reduced production of ROS, which were observed in the I/R-injured kidneys of MSC-EV-treated mice [77,78]. In line with these findings, Gu and coworkers observed preserved mitochondrial morphology in renal tubular cells of MSC-EV-treated mice [79]. They showed that miR-30 antagomirs remarkably reduced renoprotective effects of MSC-EVs, implying critical role of miR-30 in MSC-EV-based attenuation of AKI [79]. Song and colleagues further emphasized importance of MSC-derived miRNAs in renoprotection by demonstrating anti-inflammatory properties of miR-21 in alleviation of I/R-induced AKI [80]. MSC-sourced miR-21 reduced NF-κB activity in renal infiltrating DCs and suppressed their maturation [80]. Accordingly, administration of miR-21-containing MSC-EVs significantly attenuated capacity of renal DCs for production of inflammatory cytokines and reduced activation of Th1 and Th17 cell-driven inflammation in I/R-injured kidneys leading to the attenuation of AKI [80].

In addition to miR-21 and miR-30, members of the let-7 miR family, contained within MSC-EVs, have been shown to regulate multiple genes involved in apoptosis and proliferation of renal tubular epithelial cells, including CCNA2, CDC34, AURA/STK6, AURKB/STK12, E2F5, and CDK8 [81]. Moreover, MSC-derived let-7b was responsible for MSC-EV-induced generation of immunosuppressive M2 phenotype in renal macrophages [82]. Accordingly, significantly lower concentration of M1-derived inflammatory cytokines TNF-α and IL-1β were measured in I/R-injured kidneys of mice that received let-7b-containing MSC-EVs.

MSC-sourced miRNAs, particularly let-7c, targeted pro-fibrotic genes (collagen IVα1, TGF-β1 and TGFβR1) in inflamed kidneys, crucially contributing to the therapeutic effects of MSC-EVs in renal fibrosis and diabetic nephropathy [83,84,85]. In line with these findings were results obtained by Zou and colleagues who indicated that MSC-EV-dependent down-regulation of CXCL1 production was responsible for significantly decreased number of CD68+ macrophages in fibrotic kidneys of MSC-EVs-treated mice [84]. Since remarkably increased expression of MSC-sourced vascular endothelial growth factor (VEGF) was observed in the MSC-EV-treated kidneys, Zou and coworkers suggested that MSC-induced neo-angiogenesis was, in addition to MSC-EV-based immunosuppression, also responsible for beneficial effects of MSC-EVs in alleviation of renal fibrosis [86]. Since activation of MSCs with inflammatory cytokines (TNF-α and IFN-γ) significantly enhanced production of immunosuppressive and pro-angiogenic factors in MSCs-Exos [87], TNF-α and IFN-γ-priming of MSCs should be further explored as a new approach for the generation of MSC-EVs with optimal renoprotective characteristics.

## 7. MSC-EV-Based Attenuation of Autoimmune and Inflammatory Eye Disease

A large number of experimental and clinical studies demonstrated beneficial effects of MSC-Exos in the suppression of autoimmune and chronic inflammatory eye diseases [88,89,90,91,92,93,94,95]. Intravenous as well as periocular administration of MSC-Exos efficiently attenuated experimental autoimmune uveitis (EAU) [89,90]. MSC-Exos suppressed production of CCL2 and CCL21, which resulted in significantly reduced presence of Gr-1-expressing granulocytes, CD68-expressing macrophages and CD4+T cells in injured retinas [89]. While massive infiltration of inflammatory cells resulted in severe disruption of the retinal photoreceptor layers in vehicle-treated EAU mice, only little structural damage of retinal cells and few inflammatory infiltrates were observed in the eyes of MSC-Exo-treated EAU mice [90]. In addition to their effect on chemokine production, MSC-Exos inhibited antigen-presenting function of retinal-infiltrating DCs, as well. MSC-Exos significantly reduced expression of costimulatory molecules (CD40, CD80 and CD86) and MHC class II proteins on DCs, attenuating their capacity for activation of naive CD4+ T cells [90]. The transcript levels of DC-derived Th1 and Th17-related cytokines (IL-1β, IL-6 and IL-12) were significantly lower in MSC-Exos-treated animals [90]. Accordingly, remarkably reduced number of IFN-γ-producing Th1 and IL-17-producing Th17 cells, that play crucially important pathogenic role in progression of EAU, were noticed in the eyes of MSC-Exo-treated EAU mice, implying that therapeutic effects of MSC-Exos in alleviation of EAU were relied on suppression of Th1 and Th17 cell-driven inflammation [90].

Th17 cells are the main inflammatory, effector cells in dry eye disease (DED), chronic inflammatory disease of the tears and ocular surface that is manifested by symptoms of discomfort, visual disturbance, and tear film instability [91]. MSC-Exos contain a growth related oncogene (GRO), which suppresses production of Th17-inducing cytokines (IL-1β, IL-6 and IL-23) in DCs and prevent Th17 cell-driven inflammation [91,92]. In addition to GRO, MSC-sourced Indoleamine 2-3 dioxygenase (IDO) was responsible for MSC-Exo-based suppression of DC-dependent generation of Th17 cells [93,94]. Exos obtained from IDO-overexpressing MSCs down-regulated expression of co-stimulatory molecules and suppressed production of Th17-inducing cytokines in DCs, attenuating their capacity for activation of naïve T cells and generation of inflammatory Th17 cells [93,94]. Additionally, MSC-derived IDO acts as a critical molecular switch that maintains immunosuppressive phenotype of FoxP3-exspressing Tregs in inflamed tissues and prevents their re-programming into inflammatory Th17 cells [14]. Furthermore, MSC-derived IDO promotes expansion of TGFβ and IL-10-producing- immunosuppressive Tregs, contributing to the creation of immunosuppressive microenvironment in the inflamed eyes [88]. Accordingly, IDO-dependent regulation of Th17:T regulatory cells (Tregs) ratio, is also responsible for MSC-Exo-based suppression of Th17 cell driven inflammation in the eyes [88]. In line with these findings, we recently designed an ophthalmic solution (Exo-d-MAPPS), which activity was based on therapeutic effects of GRO and IDO-containing MSC-Exos [94]. Exo-d-MAPPS treatment significantly attenuated production of inflammatory cytokines in T cells and managed to alleviate dryness, grittiness, scratchiness, irritation, burning and eye fatigue in DED patients [94].

In addition to their anti-inflammatory effects, MSC-Exos promoted repair and regeneration of injured neurons in the eye [88]. Exos, obtained from bone marrow-derived MSCs, increased survival and neuritogenesis of retinal ganglion cells (RGCs) [95]. By using nerve crush model, Mead and Tomarev showed that intravitreal administration of MSC-Exos significantly reduced loss of RGCs and improved their function [95]. Therapeutic effects of MSC-Exos were relied on the delivery of miR-17-92, miR21 and miR-146 into the injured RGCs. MSC-sourced miR-17-92 and miR21 down-regulated expression of PTEN (an important suppressor of RGC axonal growth), while MSC-derived miR-146a reduced expression of epidermal growth factor receptor (involved in inhibition of axon regeneration) [95]. Importantly, beneficial effects of MSC-Exos in protection, repair and regeneration of RGCs were observed only in animals that received MSC-Exos and were not noticed after injection of fibroblasts-derived Exos [95], implying specific therapeutic potential of MSCs-Exos in regeneration of injured RGCs. Since gradual loss of RGCs is the hallmark of glaucoma, MSC-Exos represent potentially new therapeutic agents for glaucoma treatment, which efficacy should be explored in up-coming clinical trials.

## 8. Delivery of MSC-Sourced mRNAs into the Injured Cardiomyocytes Was Mainly Responsible for MSC-EVs-Based Cardioprotection

Several lines of evidence demonstrated that injection of MSC-EVs efficiently protected cardiomyocytes from ischemic injury [96,97]. By using animal model of I/R-induced myocardial injury, Lai and colleagues showed that Exos, isolated form human embryonic stem cells derived MSCs, significantly reduced infarct size and remarkably improved cardiac function in experimental animals [96]. MSC-Exos attenuated oxidative stress in I/R-injured hearts, as evidenced by greatly increased tissue levels of ATP and nicotine adenine dinucleotide and significantly decreased levels of reactive oxygen species [97]. MSC-Exos contain Parkinson protein 7/DJ-1 (DJ-1), which binds to the PARKIN protein in oxidative stress conditions, protecting the mitochondria from oxidative stress [98,99]. SinceDJ-1 protects murine heart from oxidative damage [100], MSC-sourced DJ-1 may be responsible for MSC-Exo-based modulation of oxidative balance in ischemic hearts [96]. Accordingly, Exos obtained from DJ-1-overexpressing MSCs should be explored in up-coming preclinical studies as new agents that could promote cardiac regeneration after ischemic injury. Cardioprotective effects of MSC-Exos were also relied on increased phosphorylation and activation of kinases that prevented apoptosis of injured cardiomyocytes (Akt and Glycogen synthase kinase 3 (GSK3)) and on suppression of c-Jun-N-terminal kinase, which promoted apoptosis in ischemic hearts [96]. 

Results obtained by Yu and coworkers supported the hypothesis that Akt kinase was the main intracellular target for MSC-EV-based cardioprotection [97]. They showed that Exos, obtained from Gata-4-overexpressing bone marrow derived MSCs, significantly reduced the size of ischemic lesion and restored cardiac function in the rat model of acute myocardial infarction (AMI) by activating Akt-dependent signaling pathway in injured cardiomyocytes [97]. Yu and colleagues revealed that among several MSC-Exo-containing miRNAs that regulate survival and proliferation of cardiomyocytes, miR-19a was mainly responsible for MSC-Exos-induced anti-apoptotic effects in ischemic hearts. MSC-sourced miR-19a down-regulated activation of PTEN and promoted phosphorylation and activation of Akt resulting in the up-regulation of anti-apoptotic Bcl-2 protein, resulting in reduced apoptotic loss of cardiomyocytes [97]. In line with these findings are results obtained by Wang and colleagues who demonstrated that Exos, obtained from endometrium-derived MSCs, significantly improved recovery of cardiac function after AMI by promoting Akt-dependent up-regulation of Bcl-2 activity in injured cardiomyocytes [101]. Wang et al. suggested that MSC-derived miR-21 was mainly responsible for cardioprotective effects of MSC-EVs. They demonstrated that, in addition to anti-apoptotic effects, miR21-containing MSC-Exos induced enhanced expression of vascular endothelial growth factor (VEGF) and promoted neovascularization in ischemic hearts, significantly improving cardiac function after AMI [101].

A crucially important role of MSC-sourced miRNAs for MSC-EV-based cardioprotection was confirmed by Feng and colleagues [102]. They suggested that MSC-Exo-mediated delivery of miR-22 in ischemic cardiomyocytes was mainly responsible for improved cardiac function that was noticed in MSC-Exo-treated mice with AMI [81]. Significantly reduced infarct size and cardiac fibrosis was a consequence of miR-22-dependent down-regulation of methyl-CpG-binding protein 2, epigenetic regulator, which was up-regulated in ischemic hearts [102].

It should be emphasized that, in addition to their anti-apoptotic effects, MSC-EVs also suppressed the influx of circulating leucocytes in injured hearts, contributing to the attenuation of on-going inflammation [97]. Significantly reduced release of alarmins and DAMPs from MSC-EV-treated cardiomyocytes resulted in decreased secretion of leucocyte-attracting chemokines by resident macrophages. Accordingly, after reperfusion, a significantly lower number of neutrophils, monocytes and lymphocytes infiltrated myocardium of MSC-Exo-treated animals, indicating that MSC-Exos-based suppression of inflammatory response also contributed to the enhanced repair and regeneration of injured cardiomyocytes [97]. 

## 9. Conclusions and Future Directions

MSC-EVs represent new, cell-free agents that could be used for efficient attenuation of organ-specific and systemic inflammation. Both local and systemic administration of MSC-EVs efficiently suppressed detrimental immune response in inflamed tissues and promoted survival and regeneration of injured parenchymal cells.

Through the delivery of mRNAs and miRNAs, MSC-EVs activated autophagy and/or inhibited apoptosis, necrosis and oxidative stress in injured hepatocytes, neurons, retinal cells, lung, gut and renal epithelial cells, promoting their survival and regeneration. MSC-EVs-based anti-inflammatory effects were relied on the delivery of immunoregulatory miRNAs and immunomodulatory proteins in inflammatory immune cells (M1 macrophages, DCs and Th1/Th17 cells), enabling their phenotypic conversion into anti-inflammatory and immunosuppressive cells (Table 1).

It should be noted that although experimental findings strongly suggested therapeutic potential of MSC-EVs, there is still a lot of experimental work to be done before MSC-EVs could be offered as universal human remedy for the therapy of inflammatory diseases.

MSC-EVs exhibit most of the properties of MSCs and fundamental challenges relating to MSC heterogeneity affect biological properties and therapeutic potential of MSC-EVs, as well [103]. Differences in the proliferation rate, potential for multi-lineage differentiation and immunosuppressive properties of MSCs from different sources are well-documented [104]. Furthermore, even when MSCs were obtained from the same tissue of origin, they could have prodigious donor-to-donor variation in expression of membrane markers, transcriptional and proteomic profile [104]. Aging also has a negative influence on self-renewal capacity, differentiation and immunosuppressive characteristics of MSCs, attenuating their therapeutic potential [105]. In line with these findings, several recently published studies indicated that MSC-EVs have significant tissue source and age-dependent differences in their capacity for immunosuppression and tissue regeneration [106,107,108]. Additionally, culture conditions in which MSCs were exposed may also influence the concentration of immunomodulatory factors within MSC-EVs. Significantly higher concentration of immunosuppressive cytokines were observed in EVs that were obtained from the MSCs, which were primed with inflammatory cytokines (TNF-α and IFN-γ) than in EVs that were derived from MSCs, which were grown under standard culture conditions [103]. 

Since large number of different mRNAs, miRNAs, anti-apoptotic and immunosuppressive proteins have been proposed as crucially important for beneficial effects of MSC-EVs, further experimental studies should identify the exact disease-specific MSC-sourced molecule(s) responsible for long-term protection of injured cells and/or sustained immunosuppression. Additionally, the precise dose and route of administration of MSC-EVs should be defined for each organ-specific and systemic inflammatory disease in order to prevent the development of uncontrolled immunosuppression in MSC-EVs recipients.

It should be noted that different laboratories use diverse methods to isolate and purify MSC-EVs and, accordingly, it is critical to define and standardize highly effective method for MSC-EV yields [31]. Additionally, clinical applications of MSC-EVs require their long-term use and considerable thought must be given to the preservation of their immunosuppressive potential [21]. A large number of studies demonstrated that the most convenient mode of storage for MSC-EVs remains −80 °C [109]. Nevertheless, due to the complex cold chain logistics, alternatives such as lyophilization and the incorporation of additives might be necessary to improve MSC-EV storage stability during transportation [21,109].

In summing up, due to their unique biological and immunosuppressive properties, MSC-EVs represents potentially new therapeutic agents in regenerative medicine. Once the critical questions around isolation, long-term preservation, donor and tissue source of MSC-EVs are answered, MSC-EVs will meet their full versatile potential as a new remedies in the therapy of inflammatory diseases.

## Figures and Tables

**Table 1:** Therapeutic effects of MSC-EVs in attenuation of inflammatory diseases.

Disease Model	MSC Source	Type of MSC-EVs	Target Cell	Molecular Mechanism	Therapeutic Effect	Ref. No.
DSS-induced colitis	BM	MSC-EVs	macrophage	suppression of NF-κB, iNOS-signaling pathways	generation of M2 macrophages; attenuation of colitis	[25,26,27]
DSS-induced colitis	UC	MSC-Exos	macrophage	suppression of IL-7-signaling pathway	increased secretion of IL-10; alleviation of colitis	[28]
d-GalN/LPS-induced acute liver injury	MB	MSC-Exos	hepatocytes	suppression of caspase-3-driven apoptosis	reduced apoptosis of hepatocytes; increased survival rate	[38]
Liver antigen S100-induced autoimmune hepatitis	BM	MSC-Exos	hepatocytes	inhibition of caspase-1-dependent pyroptosis	attenuation of IL-1β and IL-18-driven inflammation	[39]
Hepatic I/R injury	iPSCs	MSC-Exos	hepatocytes	increased activity of SK1	increased proliferation of hepatocytes	[42,43]
CCl4-induced liver fibrosis	UC	MSC-Exos	hepatocytes	inhibition of TGF-β1/Smad2 signaling pathway	reduced fibrosis	[44]
CCl4-induced liver fibrosis	AM	MSC-EVs	Kupffer cells	suppressed production of inflammatory cytokines	alleviated chronic liver inflammation	[46]
CCl4-induced liver fibrosis	AT	MSC-Exos	HSCs	increased expression of Beclin-1 and suppressed expression of Bcl-2	increased apoptosis and autophagy of HSCs; attenuated fibrosis	[48]
I/R-induced lung injury	BM	MSC-Exos	lung epithelial cells	suppression of caspase-3,-8 and -9	Inhibition of apoptosis; alleviation of lung injury	[50]
*E. coli*-induced pneumonia	BM	MSC-EVs	neutrophils; monocytes	increased synthesis of LTB4	increased phagocytosis; reduced pneumonia	[53]
Bleomycin-induced lung fibrosis	BM	MSC-Exos	alveolar macrophages	suppressed production of TGF-β1	reduced deposition of collagen in the lungs	[57]
Aspergillus protease antigen-induced lung inflammation	AT	MSC-EVs	lung DCs	reduced expression of co-stimulatory molecules and increased production of IL-10	alleviation of Th2 cell-driven lung inflammation	[58]
AβPP/PS1 transgenic mice	UC	MSC-Exos	microglia	polarization towards M2 phenotype	increased spatial learning and memory; attenuated AD	[62]
primary hippocampal cultures exposed to Aβ	WJ	MSC-EVs	hippocampal neurons	catalase-dependent attenuation of oxidative stress-induced injury	prevention of neuronal damage	[64]
TMEV-induced MS	AT	MSC-EVs	Microglia; CD4+T cells	suppressed production of inflammatory cytokines	alleviated neuroinflammation	[65]
L5/6 spinal nerve ligation	UC	MSC-Exos	neurons	delivery of neurotrophic factors	better recovery from nerve ligation-induced injury	[66]
Cerebral I/R-induced injury	AT	MSC-Exos	neurons	induction of autophagy and suppression of apoptosis	prevention of neural cell death	[67]
CDDP-induced AKI	BM	MSC-EVs	renal tubular cells	increased proliferation and suppressed apoptosis	alleviation of AKI	[71]
I/R-induced AKI	BM	MSC-EVs	renal tubular cells	increased proliferation and suppressed apoptosis	reduced impairment of renal function	[74]
CDDP-induced AKI	UC	MSC-Exos	PTECs	induction of autophagy	attenuation of AKI	[75,76]
I/R-induced AKI	WJ	MSC-EVs	renal tubular cells	attenuation of oxidative stress	alleviation of AKI	[77]
I/R-induced renal injury	WJ	MSC-EVs	macrophages	suppressed CXCL1-dependent influx of monocytes in injured kidneys	improvement of renal function and abrogation of renal fibrosis	[84]
EAU	BM	MSC-EVs	DCs	reduced expression of costimulatory molecules and reduced production of Th17-related cytokines	attenuated Th17-driven inflammation in the eyes	[90]
Optic nerve crush	BM	MSC-Exos	RGCs	increased neuritogenesis	increased survival of RGCs	[95]
I/R-induced myocardial injury	ESCs	MSC-Exos	cardiomyocytes	attenuated oxidative stress	reduced infarct size and improved cardiac function	[96]
AMI	BM	MSC-Exos	cardiomyocytes	activation of Akt-dependent signaling pathway	reduced infarct size and improved cardiac function	[97]
AMI	EM	MSC-Exos	cardiomyocytes	increased production of VEGF	increased neovascularization in ischemic hearts	[101]

Abbreviations: Dextran sulfate sodium (DSS); bone marrow (BM); umbilical cord (UC); D-Galactosamine/Lipopolysaccharide (D-GalN/LPS); menstrual blood (MB); ischemia-reperfusion (I/R); induced pluripotent stem cells (iPSC); sphingosine kinase (SK1); carbon tetrachloride (CCl4); amnion (AM); adipose tissue (AT); hepatic stellate cells (HSCs); *Escherichia coli* (*E. coli*); leukotriene B4 (LTB4); dendritic cells (DCs); amyloid-β peptide (Aβ); Alzheimer’s disease (AD); Wharton’s jelly (WJ); Theiler’s murine encephalomyelitis virus (TMEV); multiple sclerosis (MS); cisplatin (CDDP); acute kidney injury (AKI); proximal tubular epithelial cells (PTEC); Experimental autoimmune uveitis (EAU); retinal ganglion cells (RGCs); embryonic stem cells (ESCs); acute myocardial infarction (AMI); endometrium (EM); vascular endothelial growth factor (VEGF).
